# CRISPR-Cas9 treatment partially restores amyloid-β 42/40 in human fibroblasts with the Alzheimer’s disease *PSEN1* M146L mutation

**DOI:** 10.1016/j.omtn.2022.03.022

**Published:** 2022-03-28

**Authors:** Evangelos Konstantinidis, Agnieszka Molisak, Florian Perrin, Linn Streubel-Gallasch, Sarah Fayad, Daniel Y. Kim, Karl Petri, Martin J. Aryee, Ximena Aguilar, Bence György, Vilmantas Giedraitis, J. Keith Joung, Vikram Pattanayak, Magnus Essand, Anna Erlandsson, Oksana Berezovska, Martin Ingelsson

**Affiliations:** 1 Department of Public Health and Caring Sciences, Molecular Geriatrics, Rudbeck Laboratory, Uppsala University, Uppsala, Sweden; 2 Department of Neurology, Massachusetts General Hospital, Memory Disorders Unit, Harvard Medical School, Charlestown, MA, USA; 3 Molecular Pathology Unit, Massachusetts General Hospital, Charlestown, MA, USA; 4 Center for Cancer Research, Massachusetts General Hospital, Charlestown, MA, USA; 5 Center for Computational and Integrative Biology, Massachusetts General Hospital, Charlestown, MA, USA; 6 Department of Pathology, Harvard Medical School, Boston, MA, USA; 7 Department of Biostatistics, Harvard T.H. Chan School of Public Health, Boston, MA, USA; 8 Institute of Molecular and Clinical Ophthalmology Basel, Basel, Switzerland; 9 Department of Ophthalmology, University of Basel, Basel, Switzerland; 10 Department of Immunology, Genetics and Pathology, Uppsala University, Uppsala, Sweden; 11 Krembil Brain Institute, University Health Network, Toronto, ON, Canada; 12 Department of Medicine and Tanz Centre for Research in Neurodegenerative Diseases, University of Toronto, Toronto, ON, Canada

**Keywords:** MT:: RNA/DNA editing, Alzheimer’s disease, presenilin 1, amyloid-β, fibroblasts, CRISPR-Cas9, gene editing, protein conformation, off-target effects

## Abstract

Presenilin 1 (PS1) is a central component of γ-secretase, an enzymatic complex involved in the generation of the amyloid-β (Aβ) peptide that deposits as plaques in the Alzheimer’s disease (AD) brain. The M146L mutation in the PS1 gene (*PSEN1*) leads to an autosomal dominant form of early-onset AD by promoting a relative increase in the generation of the more aggregation-prone Aβ42. This change is evident not only in the brain but also in peripheral cells of mutation carriers. In this study we used the CRISPR-Cas9 system from *Streptococcus pyogenes* to selectively disrupt the *PSEN1*^*M146L*^ allele in human fibroblasts. A disruption of more than 50% of mutant alleles was observed in all CRISPR-Cas9-treated samples, resulting in reduced extracellular Aβ42/40 ratios. Fluorescence resonance energy transfer-based conformation and western blot analyses indicated that CRISPR-Cas9 treatment also affects the overall PS1 conformation and reduces PS1 levels. Moreover, our guide RNA did not lead to any detectable editing at the highest-ranking candidate off-target sites identified by ONE-seq and CIRCLE-seq. Overall, our data support the effectiveness of CRISPR-Cas9 in selectively targeting the *PSEN1*^*M146L*^ allele and counteracting the AD-associated phenotype. We believe that this system could be developed into a therapeutic strategy for patients with this and other dominant mutations leading to early-onset AD.

## Graphical abstract

**Figure:**
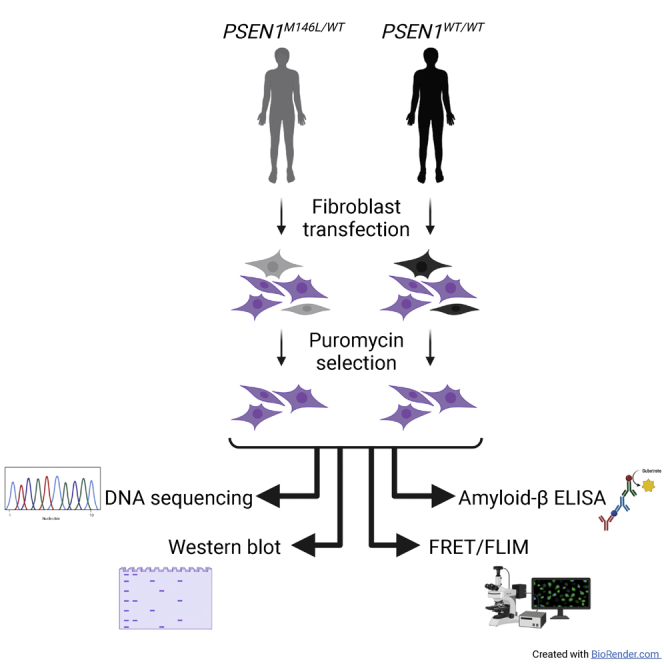

## Teaser

As a novel approach for treatment of early-onset familial Alzheimer’s disease, CRISPR-Cas9-mediated disruption of *PSEN1* in human fibroblasts with the pathogenic *PSEN1* M146L mutation can partially restore the disease-associated amyloid-β 42/40 increase, as well as reduce presenilin 1 levels.

## Introduction

Alzheimer’s disease (AD) is the most common neurodegenerative disease and affects more than 50 million people worldwide.^1^ Sporadic, late-onset AD accounts for more than 95% of the total number of patients, whereas familial early-onset AD (EOAD) represents less than 5% of the cases.^2^ Early-onset disease forms are associated with symptoms already in the fifth and sixth decades of life and are caused by autosomal dominant mutations in *APP*, *PSEN1* and *PSEN2*, genes encoding for amyloid precursor protein (APP), presenilin 1 (PS1), and presenilin 2 (PS2), respectively. The pathogenic effects of all mutations converge toward an increased generation or altered conformation of amyloid-β (Aβ), the peptide that accumulates as plaques in the AD brain.

Mutations in *PSEN1* are the most common cause of familial EOAD and generally lead to an earlier age of onset compared with mutations in the other two genes.^3^ To date, more than 300 *PSEN1* mutations have been described, with most of them increasing the generation of the aggregation-prone Aβ42 in relation to Aβ40.^4^^,^^5^ Presenilin 1 is the catalytic domain of the γ-secretase (GS) complex that is responsible for the final cleavage step of APP in the amyloidogenic pathway and the subsequent release of Aβ peptides of varying lengths.^6^^,^^7^

The underlying reasons for the shift in the Aβ42/40 ratio are not fully understood, although studies have indicated a partial loss of function^8^^,^^9^ and/or conformational changes with an altered substrate recognition of PS1^10^ as related mechanisms. Moreover, *PSEN1* mutations have been reported to result in a tighter conformation of PS1 that brings its C- and N-termini in closer proximity to each other and thereby affects APP recognition and/or cleavage by favoring the production of Aβ42 over Aβ40.^11^^,^^12^

The *PSEN1* M146L (A > C) mutation was first identified in 1995, together with the cloning of the *PSEN1* locus on chromosome 14, in a family in southern Italy (FAD4) and results in EOAD with an average onset at 43 years of age.^13^ Carriers of this mutation display an autosomal dominant pattern of inheritance.^14^ The effects on APP processing by *PSEN1* M146L can also be detected outside of the brain. For example, an elevated Aβ42/40 ratio can be measured both in fibroblasts and induced pluripotent stem cell (iPSC)-derived neurons from mutation carriers.^15^

Advances in gene editing have made it possible to target disease-causing mutation-carrying alleles with high selectivity.^16^ The CRISPR-Cas9 system consists of a guide RNA (gRNA) of 19–21 bp directed against the sequence of interest together with an RNA-guided CRISPR-associated (Cas)9 nuclease that induces precise double-strand DNA breaks.^17^^,^^18^ The presence of a protospacer adjacent motif (PAM) site downstream of the target sequence is essential for Cas9 recognition and its subsequent cleavage.^19^ The preferred PAM site for *Streptococcus pyogenes* Cas9 (SpCas9) is 5′-*NGG*-3′.^20^^,^^21^ Upon the induction of a double-strand break, the cell attempts to repair the damage either by non-homologous end joining (NHEJ) or by homology-directed repair.^22^ In NHEJ, DNA insertions or deletions (indels) at the targeted locus can affect gene expression either by changing the coding frame of the protein via nonsense-mediated decay (NMD) through premature stop codons or by disrupting the effective binding of transcription factors.^23^^,^^24^

The NHEJ approach was recently used by us to selectively disrupt the Swedish mutation in the *APP* gene that leads to increased production of Aβ.^25^ The treatment was shown to correct the mutational effect on APP processing by normalizing total Aβ levels in AD patient fibroblasts. In addition, targeting of cultured primary neurons from transgenic APPswe mice and hippocampal neurons in living mice resulted in specific disruption of the mutated allele.

We here present a CRISPR-Cas9-based treatment strategy against the *PSEN1* M146L mutation. We aimed to evaluate whether specific disruption of the *PSEN1*^*M146L*^ allele in *PSEN1*^*M146L/WT*^ fibroblasts leads to a normalized Aβ42/40 ratio and whether this change has any effect on the altered PS1 conformation associated with the mutation.

## Results

### CRISPR-Cas9-mediated disruption of *PSEN1* M146L in human fibroblasts

**Figure 1:**
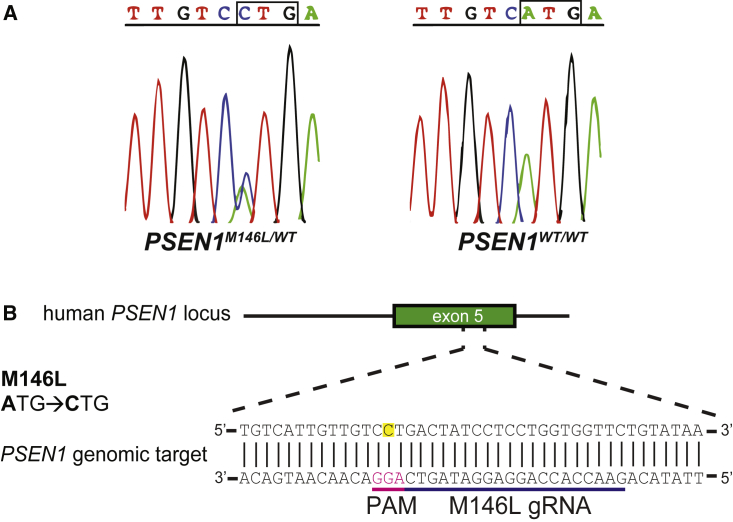
Sequencing traces of the *PSEN1* locus and gRNA design (A) All human fibroblast samples were sequenced for the presence of the *PSEN1* M146L (A > C) mutation in exon 5 of the *PSEN1* gene. The results from one *PSEN1* M146L mutated and one non-mutated subject are shown. (B) This mutation creates a 5′-*NGG*-3′ PAM site on the reverse DNA strand that allows for generation of the 20-bp-long allele-specific M146L gRNA.

We analyzed fibroblasts from six subjects carrying the *PSEN1* M146L mutation, as well as fibroblasts from two healthy controls from the same family together with two unrelated controls (Table S1). Mutant and wild-type fibroblasts were sequenced to verify the presence of the A-to-C mutation (Figure 1A). This mutation offers a unique opportunity for gRNA design, as it creates a PAM site suitable for SpCas9 (5′-*NGG*-3′) in the reverse DNA strand that is not present in the wild-type allele (Figure 1B). Although SpCas9 seems to recognize non-canonical PAM sites as well, studies suggest a very low preference for a 5′-*NTG*-3′ PAM site like the one that exists in the wild-type (*PSEN1*^*WT*^) allele.^26^ Therefore, we hypothesized that the 20-bp-long M146L gRNA that we designed would target the *PSEN1*^*M146L*^ allele while leaving the *PSEN1*^*WT*^ allele intact.

**Figure 2:**
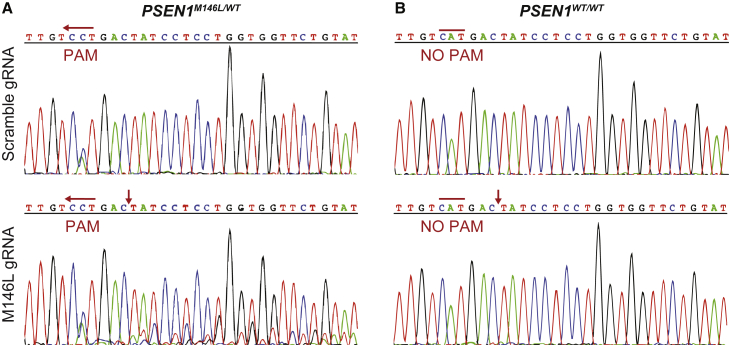
CRISPR-Cas9-mediated disruption of the *PSEN1*^*M146L*^ allele in human fibroblasts (A) Expression of SpCas9 and M146L gRNA in *PSEN1*^*M146L/WT*^ fibroblasts was disruptive and created indels (as indicated by background peaks downstream of the cut site, red arrows). (B) No indel formation was evident in *PSEN1*^*WT/WT*^ fibroblasts that lack the crucial PAM site.

Human fibroblasts carrying the *PSEN1* M146L mutation (*PSEN1*^*M146L/WT*^) (n = 6) as well as age-matched controls (*PSEN1*^*WT/WT*^) (n = 4) were transfected with a plasmid vector that expresses SpCas9 together with either the M146L-specific gRNA or a scramble (control) gRNA that does not recognize any sequence in the human genome. Sanger sequencing of DNA from SpCas9 + M146L gRNA-treated *PSEN1*^*M146L/WT*^ fibroblasts showed robust, target-specific indel formation, indicated by additional peaks around the intended cleavage site (Figure 2A). In contrast, there was no disruption in the *PSEN1*^*WT/WT*^ fibroblasts treated with SpCas9 + M146L gRNA, which verifies the selectivity of our approach (Figure 2B). Furthermore, no indel formation was evident in the fibroblasts treated with the scramble gRNA sequence (Figures 2A and 2B).

**Figure 3:**
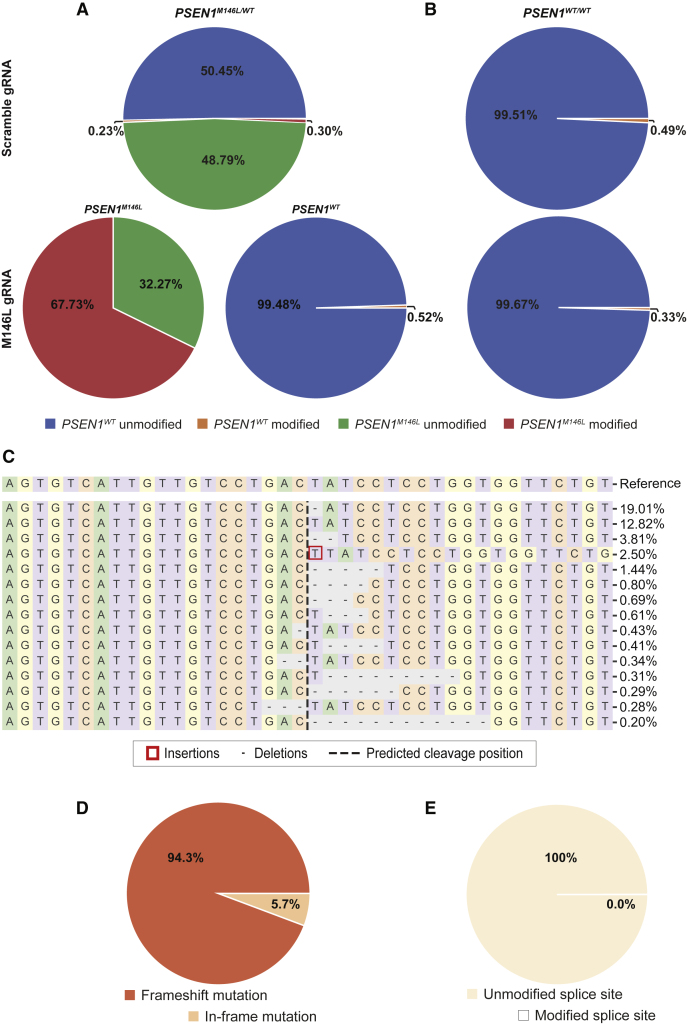
CRISPR-Cas9 can selectively target the *PSEN1*^*M146L*^ allele in human fibroblasts (A and B) NGS analysis of the *PSEN1* allele from CRISPR-Cas9-treated *PSEN1*^*M146L/WT*^ and *PSEN1*^*WT/WT*^ fibroblasts with CRISPResso2. Alignment and editing frequency of reads as determined by the percentage and number of sequence reads showing unmodified and modified *PSEN1* alleles from CRISPR-Cas9-treated *PSEN1*^*M146L/WT*^ and *PSEN1*^*WT/WT*^ fibroblasts. The M146L gRNA resulted in indel formation in the *PSEN1*^*M146L*^ but not in the *PSEN1*^*WT*^ allele (indel formation is depicted separately for the different alleles of the *PSEN1*^*M146L/WT*^ fibroblasts). (C) Visualization of the distribution of the 15 most frequently identified alleles around the cleavage site. Nucleotides are indicated by unique colors (A = green; C = red; G = yellow; T = purple). Red rectangles highlight inserted sequences. Horizontal dashed lines indicate deleted sequences. The vertical dashed line indicates the predicted cleavage site. The most common modification was a single base pair deletion. (D) Frameshift analysis of coding sequence reads affected by modifications (unmodified reads are excluded from this analysis). (E) Predicted impact on splice sites. Modified splice site refers to a read in which either of the two intronic positions adjacent to exon junctions is disrupted.

We assessed the percentage of allelic disruption with next-generation sequencing (NGS) followed by analysis with CRISPResso2.^27^ The results showed up to 67.73% disruption of the targeted *PSEN1*^*M146L*^ allele in *PSEN1*^*M146L/WT*^ fibroblasts while there was no disruption in the *PSEN1*^*WT*^ allele, as suggested by the comparable percentage of *PSEN1*^*WT*^ in M146L and scramble gRNA-treated fibroblasts (Figure 3A and Table S2). To further validate the specificity of our gRNA, we analyzed DNA from CRISPR-Cas9-treated *PSEN1*^*WT/WT*^ fibroblasts and could not identify any indels (Figure 3B). However, we observed rare indels in the *PSEN1*^*WT*^ allele, off the cleavage site, both in *PSEN1*^*M146L/WT*^ and *PSEN1*^*WT/WT*^ fibroblasts upon treatment with either M146L or scramble gRNAs (Figure S1).

Next, we evaluated the CRISPR-induced changes in *PSEN1*^*M146L/WT*^ fibroblasts and found that the majority of modifications are represented by deletions around the cleavage site (95%). A single base pair deletion was the most common event, followed by a 2-bp deletion and a 1-bp insertion (Figure 3C). Frameshift analyses of the coding sequence reads (*PSEN1* exon 5) confirmed that a majority of the indels resulted in frameshift mutations (94.3%), whereas only 5.7% led to in-frame mutations (Figure 3D). Given the central position of the M146L mutation within exon 5 as well as the target sequence of the M146L gRNA, we also evaluated the effect of the treatment on potential splice sites. We did not find any evidence of modifications within the exon-intron junctions on either side of exon 5 (Figure 3E).

### CRISPR-Cas9 treatment partially restores the extracellular Aβ42/40 ratio

**Figure 4:**
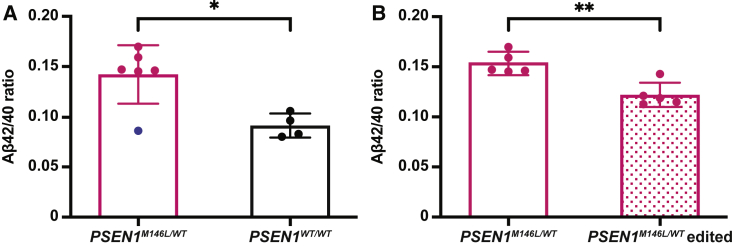
CRISPR-Cas9 treatment reduces the Aβ42/40 ratio in conditioned medium of treated *PSEN1*^*M146L/WT*^ human fibroblasts (A) Consistent with previous findings, the ratio of Aβ42/40 was elevated in *PSEN1*^*M146L/WT*^ fibroblasts (n = 6) compared with *PSEN1*^*WT/WT*^ (n = 4) (blue data point represents a *PSEN1*^*M146L/WT*^ fibroblast sample that did not display the pathological phenotype). (B) *PSEN1*^*M146L/WT*^ fibroblasts before and after transfection with SpCas9 and M146L gRNA (n = 5). Results are presented as mean±SD; unpaired two-tailed t test; ∗∗p < 0.01, ∗p < 0.05.

We next investigated the effect of disrupting the *PSEN1*^*M146L*^ allele on the extracellular Aβ42/40 ratio in CRISPR-Cas9-treated *PSEN1*^*M146L/WT*^ fibroblasts. Non-treated *PSEN1*^*M146L/WT*^ fibroblasts (n = 6) displayed the expected increase in Aβ42/40 ratio in the conditioned medium, compared with non-treated *PSEN1*^*WT/WT*^ (n = 4) (Figure 4A).^15^ One of the *PSEN1*^*M146L/WT*^ fibroblast samples (blue data point) did not display the pathological phenotype of an increased Aβ42/40 ratio and was therefore excluded from further analyses. After treatment with M146L gRNA, *PSEN1*^*M146L/WT*^ edited fibroblasts displayed a significant reduction of the Aβ42/40 ratio (Figure 4B). However, the ratio was still higher than that for the *PSEN1*^*WT/WT*^ fibroblasts, whose ratio did not change after the treatment (Figure S2).

Given the numerous substrates that PS1 can cleave, we also evaluated the effect of *PSEN1* CRISPR-Cas9-mediated disruption on cell viability and ATP levels. Our data suggest that there were no differences between treated and non-treated samples (Figure S3). We also sought to evaluate levels of Notch1 intracellular domain in cell lysates of *PSEN1*^*M146L/WT*^ and *PSEN1*^*WT/WT*^ fibroblasts but were unable to detect it in either.

### CRISPR-Cas9 treatment affects pathological PS1 conformation and lowers PS1 levels

**Figure 5:**
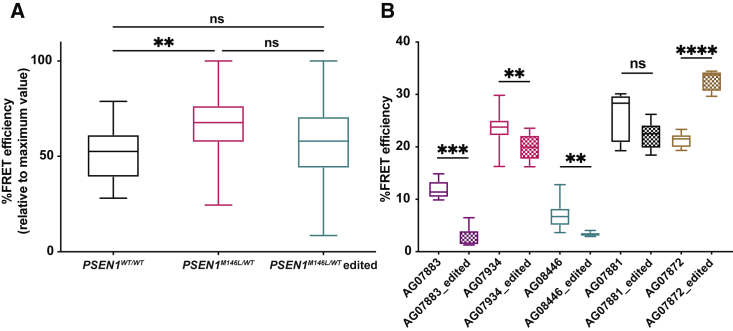
CRISPR-Cas9 treatment changes %FRET efficiency in *PSEN1*^*M146L/WT*^ fibroblasts (A) *PSEN1*^*M146L/WT*^ fibroblasts (n = 5) display a significant increase in %FRET efficiency compared with *PSEN1*^*WT/WT*^ fibroblasts (n = 2) that appears to decrease after CRISPR-Cas9 treatment (*PSEN1*^*M146L/WT*^ edited). Results are presented as median (min ÷ max) values of %FRET efficiency normalized to the highest value measured; Kruskal-Wallis one-way ANOVA with Dunn’s multiple comparison as post hoc test; n = 1 (∼90 cells), n = 2 (∼180 cells), and n = 2 (∼180 cells) independent experiments for *PSEN1*^*WT/WT*^, *PSEN1*^*M146L/WT*^, and *PSEN1*^*M146L/WT*^ edited, respectively. (B) Most individual *PSEN1*^*M146L/WT*^ fibroblast samples display a significant reduction in %FRET efficiency after CRISPR-Cas9 treatment except for AG07872, which shows an increase. Results are presented as median (min ÷ max) values of %FRET efficiency; unpaired two-tailed t test; ∗∗∗∗p < 0.0001, ∗∗∗p < 0.001, ∗∗p < 0.01, ∗p < 0.05, ns = not significant.

Next, we investigated whether the effect of the CRISPR-Cas9 treatment on the Aβ42/40 ratio could be related to a molecular alteration of PS1.^11^ Thus, we subjected the CRISPR-Cas9-treated *PSEN1*^*M146L/WT*^ fibroblasts to fluorescence resonance energy transfer/fluorescence lifetime imaging (FRET/FLIM) to seek whether targeting of *PSEN1*^*M146L*^ could rescue such a conformational shift. The *PSEN1*^*M146L/WT*^ (n = 5) and *PSEN1*^*WT*^ (n = 2) fibroblasts that had been treated with M146L gRNA were double-stained by fluorophore-conjugated antibodies against the C- and N-terminal parts of PS1. The calculated %FRET efficiency (*E*_FRET_) attested to whether the PS1/γ-secretase was in a pathogenic tight (high *E*_FRET_) or in a relaxed, wild-type-associated, open (lower *E*_FRET_) conformation. We found that the %FRET efficiency was significantly higher in *PSEN1*^*M146L/WT*^ compared with *PSEN1*^*WT/WT*^ control fibroblasts and that the CRISPR-Cas9 treatment seemed to alter *E*_FRET_ in the familial AD (FAD) mutant PS1 cells, although the analysis did not reveal any significant group differences between the treated and non-treated cells (Figure 5A). However, of the five *PSEN1*^*M146L/WT*^ treated fibroblast samples analyzed by FRET/FLIM, three displayed a significant decrease, one a trend for decrease, and one a significant increase in %FRET efficiency (Figure 5B).

We also analyzed the levels of PS1 C- and N-terminal fragments in lysates from CRISPR-Cas9-treated and control *PSEN1*^*M146L/WT*^ and *PSEN1*^*WT/WT*^ samples (Figure S4). We detected a significant reduction in both C- and N-terminal fragments of CRISPR-Cas9-treated *PSEN1*^*M146L/WT*^ samples, while no reduction was evident in *PSEN1*^*WT/WT*^ samples (Figure S4B).

In an attempt to find a genetic explanation for the observed difference in the PS1 conformational change, we performed additional DNA analyses of the five *PSEN1*^*M146L/WT*^ samples. No additional pathogenic mutations in the *PSEN1/2*, *APP*, *MAPT*, or *TREM2* genes were identified. Of note, the AG07872 sample carried the apolipoprotein E (*APOE*) ε2/3 genotype, whereas all other samples carried the *APOE* ε3/3 genotype. Overall, the FRET/FLIM data suggest that disruption of the *PSEN1*^*M146L*^ allele affects the conformation of PS1, in most cases by partially restoring the distance between its C- and N-terminal parts of the protein.

### Analysis of off-target effects

To evaluate off-target editing in *PSEN1*^*M146L/WT*^ fibroblasts, we performed oligonucleotide enrichment and sequencing (ONE-seq)^28^ using a synthetic human genomic library, based on homology to the *PSEN1* M146L gRNA sequence, that was subsequently treated with SpCas9 and M146L gRNA to nominate potential off-target sites. We also performed circularization for *in vitro* reporting of cleavage effects by sequencing (CIRCLE-seq).^29^ We compiled a list of the top ten off-target loci by considering sites that were nominated by both assays (Table 1).

**Table 1:** List of potential off-target loci as detected by ONE-seq and CIRCLE-seq

	Chromosome	Left hg19 coordinate	Right hg19 coordinate	Candidate off-target site	ONE-seq score	CIRCLE-seq read count
Off1	19	35402517	35402540	GggaCACCAGGAaGATAGTCAGG	1.67	486
Off2	5	77208877	77208900	ccACCACCgGGAGGATAaTCAGG	1.30	280
Off3	6	37584361	37584384	GgACCACCAGGAGGtaAGTCCGG	0.82	196
Off4	5	139044584	139044607	cAgtgACCAGGAGGATAGTCTGG	0.77	280
Off5	5	138720545	138720568	tAgaCACCAGGAGGAaAGTCAGG	0.37	248
Off6	4	26495731	26495754	GAAtCtCCAGGAGGAaAGTCTGG	0.36	314
Off7	9	128139428	128139451	cAACCACCAGcAGGAaAGTCTGG	0.19	302
Off8	2	233408297	233408320	tcACCACCAGGAGGAagGTCAGG	0.06	410
Off9	3	133118656	133118679	ccACCACCAGcAGaATAaTCTGG	0.03	740
Off10	2	2517636	2517659	ccACCACCtGGAaGATAGcCTGG	0.02	948

**Figure 6:**
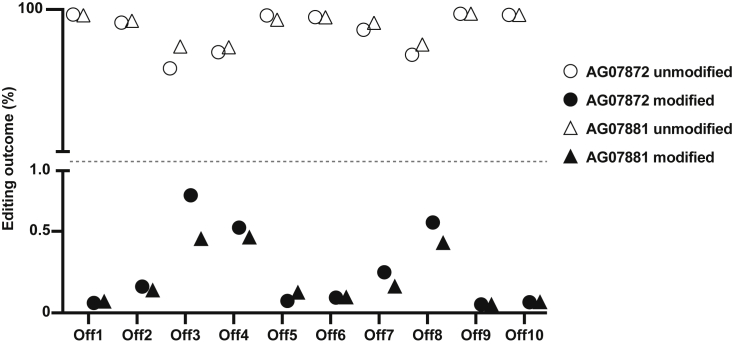
NGS analysis of the top ten predicted off-target loci Analysis by NGS of the top ten predicted off-target loci in CRISPR-Cas9-treated *PSEN1*^*M146L/WT*^ fibroblasts with CRISPResso2 (n = 2). Alignment and editing frequency of reads as determined by the percentage of sequence reads showing modifications in ten different genomic areas with similar sequences to the M146L gRNA (Off1–Off10). The M146L gRNA did not lead to indel formation in any of the top ten predicted off-target loci (modification <1%).

The NGS analysis of the top ten potential off-target loci indicated less than 1% of editing for all nominated sites, suggesting that the M146L gRNA is not only able to distinguish between the wild-type and mutant *PSEN1* alleles but is also specific in recognizing the on-target sequence (Figure 6).

## Discussion

At present, there is no cure for AD and current treatments are palliative rather than targeting the underlying causes of the disease. Acetylcholinesterase inhibitors and *N*-methyl-D-aspartate receptor antagonists have been proven to relieve some of the cognitive symptoms^30^ and delay placement in nursing homes.^31^ As for therapies directed against the underlying disease pathology, in June 2021 the US Food and Drug Administration (FDA) approved aducanumab (Aduhelm) as the first ever immunotherapy treatment designed against toxic forms of Aβ.^32^ Another monoclonal antibody, lecanemab, targeting toxic Aβ protofibrils, has shown promise in a phase II clinical trial.^33^

Inhibitors of the secretase complexes involved in APP processing have also been extensively studied as potential therapeutic targets. However, the γ-secretase inhibitors were found to cause severe side effects in late-stage clinical trials, presumably due to the large number of substrates that this enzyme recognizes.^34^^,^^35^ In this study we aimed to specifically target presenilin 1, the catalytic subunit of the γ-secretase complex, at the genetic level, thereby avoiding any issues with substrate specificity.

Gene therapies against several neurological diseases have emerged in the past 5 years. In 2019 the FDA approved the first ever gene therapy for a neurological disorder, spinal muscular atrophy,^36^ and in 2020 the first gene-editing clinical trial where CRISPR components were administered directly to patients entered its phase I stage against the rare eye disorder Leber’s congenital amaurosis.^37^ More recently, a clinical trial using CRISPR-Cas9 against hereditary transthyretin amyloidosis also entered the phase I stage in the first clinical trial for gene therapies of amyloid-related diseases.^38^ With regard to AD, no studies in humans have been initiated, but systemic delivery of CRISPR components in transgenic human *APP* mice showed robust indel formation in the brain, reduction in Aβ-associated pathologies, and improvement in cognitive performance.^39^

Early-onset AD is caused by mutations in autosomal dominant genes, making it an ideal candidate for gene-editing strategies. Several studies have pointed out how various *PSEN1* and *APP* mutations lead to pathogenic phenotypes in isogenic human cell lines and how substitution of the mutant with the wild-type allele can reverse that pathology.^40^, ^41^, ^42^ Most *PSEN1* mutations, such as *PSEN1* M146L, lead to increased production of the more aggregation-prone Aβ42 that is known to form Aβ plaques in the AD brain. By selectively targeting such mutations, we might be able to counteract the increase in Aβ42/40 ratio and thereby prevent the progression of Aβ pathology. Previously, it has been shown that CRISPR-Cas9-mediated correction of neurons derived from human *PSEN2* N141I fibroblasts resulted in normalization of the Aβ42/40 ratio as well as rescue of the associated electrophysiological deficits.^43^ In our current study we describe an allele-specific approach for targeting the *PSEN1*^*M146L*^ allele in human fibroblasts with the CRISPR-Cas9 system that is based on disruption rather than correction of the mutated allele. The M146L mutation leads to the generation of a novel PAM site in the sequence of the mutated allele that allowed us to design a gRNA that selectively disrupts the *PSEN1*^*M146L*^ allele while leaving the *PSEN1*^*WT*^ allele intact. Although the NHEJ pathway results in random indel formation, it can be very useful for targeting autosomal dominant mutations where disruption of the mutated allele could potentially suffice for phenotype restoration.^25^ The precise targeting of the mutated over the wild-type allele is of great significance, and gRNA sequences must be thoroughly tested and validated. We showed that the most common indel was a 1-bp deletion in the *PSEN1*^*M146L*^ allele at the SpCas9 cleavage site, which in turn results in a frameshift deletion in exon 5 that could alter gene expression levels through the NMD process. In fact, while the majority of indels led to frameshift mutations, there was a small percentage that led to in-frame mutations. Further analyses of these indels are required to better understand their role in potentially introducing novel *PSEN1* mutants. Nevertheless, we were able to demonstrate a significant reduction of the Aβ42/40 ratio in conditioned media from CRISPR-Cas9-treated fibroblasts from mutation carriers.

Although the Aβ42/40 ratio was restored, it did not reach the levels of the wild-type control samples. We hypothesize that this was due to the heterogeneity of the treated samples. The fibroblasts could not be clonally expanded and did not allow us to sort them as single cells after CRISPR-Cas9 treatment. Therefore, we had to analyze the editing efficiencies in a pool of cells that had undergone different gene-editing events. While this setup did not result in a complete restoration of the AD-related phenotype, we believe that it better resembles the real-life situation where not all of the cells will have the exact same editing results.

It has been proposed that an altered conformation of PS1 may explain why *PSEN1* mutations cause a pathological increase in Aβ42. Interestingly, a recent study has confirmed that the Met146 amino acid is at the very center of the recognition pocket where PS1 binds APP, which may explain the tighter conformation of PS1 in *PSEN1* M146L iPSC-derived neurons.^11^^,^^44^ Here, we showed that the mutation-related PS1 conformation was also evident in human *PSEN1*^*M146L/WT*^ fibroblasts by using FRET/FLIM to measure the proximity between the C- and N-termini of PS1. Although it could not be shown consistently, most of the treated cell samples restored the wild-type PS1 conformation. Moreover, western blot quantification of the PS1 C- and N-terminal fragments revealed significantly lower levels of both fragments after CRISPR-Cas9 treatment in cell lysates of *PSEN1*^*M146L/WT*^ but not *PSEN1*^*WT/WT*^ fibroblasts. Hence, we hypothesize that disruption of the *PSEN1*^*M146L*^ allele decreases the expression of mutated PS1 while allowing for a larger proportion of wild-type PS1 to participate in the formation of the γ-secretase complex.

The importance of the PAM site in the CRISPR-Cas9 system has been highlighted in several publications. SpCas9 in particular appears to also recognize non-canonical PAM sites, albeit with lower affinity.^26^ Nevertheless, we did not observe any cleavage activity in the wild-type *PSEN1* allele when using the M146L gRNA, supporting the preference of SpCas9 to the 5′-*NGG*-3′ PAM site over the non-canonical 5′-*NTG*-3′ PAM site. Whereas SpCas9 can also tolerate mismatches in the gRNA sequence, especially in the base pairs at the distal 3′ end of the PAM site, increasing the probability of off-target activity,^45^ novel SpCas9 variants offer increased binding specificity that could potentially minimize non-intended double-strand DNA breaks.^46^^,^^47^ Currently there are more than ten Cas9 nucleases that have been identified in different bacterial strains, each with its own unique PAM site.^48^, ^49^, ^50^, ^51^, ^52^, ^53^, ^54^, ^55^ Further engineering of the existing enzymes, in combination with identification of novel variants, will greatly expand the number of pathogenic mutations and, more generally, the genomic areas of interest that can be precisely targeted.

Recently, a study utilized a combination of Cas9 and an engineered reverse transcriptase, known as prime editing,^56^ to introduce the rare, AD-protective Icelandic *APP* mutation in human cell lines and study its behavior.^57^ Following their successful application of this novel tool, the authors also demonstrated how it can be adapted to precisely target and replace pathogenic mutations, such as the FAD-causing London *APP* mutation, thereby providing yet another treatment strategy for FAD.^57^

Off-target effects are one of the main concerns for any CRISPR-Cas9 treatment strategy. *In silico* off-target prediction algorithms^58^ can identify sites with closely matched sequences to used gRNAs, but generally need to be complemented by experimental off-target nomination and validation methods^59^ to bridge the differences between mathematic calculations and actual cellular events.^60^ In this study we adopted two different off-target detection methods, one recently developed (ONE-seq) and one previously established (CIRCLE-seq). We generated a list of the top ten potential off-target loci by combining data from both detection methods to focus on the set of off-target sites most likely to be affected. However, none of the investigated off-target areas displayed any significant (>1%) off-target editing events.

Overall, our study indicates that the CRISPR-Cas9 method can be used to selectively disrupt the EOAD-causing *PSEN1*^*M146L*^ allele and partially restore the elevated Aβ42/40 ratio that drives the pathogenesis in mutation carriers. We believe that this system could be further developed into a functioning therapy against early-onset forms of AD caused by this and other pathogenic mutations in disease-associated genes.

## Materials and methods

### Human fibroblasts

Human *PSEN1*^*M146L/WT*^ fibroblasts were obtained from The NIGMS Human Genetic Cell Repository at the Coriell Institute for Medical Research (n = 6) (Camden, NJ, USA), whereas non-mutated control fibroblasts from subjects of the same or another family were obtained from the Coriell Institute (n=2) and from the Uppsala Biobank (n=2) (Uppsala, Sweden) (Table S1) and grown in 75 cm^2^ cell-culture flasks (Sarstedt, Nümbrecht, Germany) in Dulbecco’s modified Eagle’s medium (low glucose, pyruvate, no glutamine, no phenol red) with 10% fetal bovine serum (FBS), 1% penicillin-streptomycin (10,000 U/mL), and 1% GlutaMAX supplement (100×) (all from Gibco, Thermo Fisher Scientific, Waltham, MA, USA). The study was approved by the Regional Ethical Review Board of Uppsala, Sweden, and the Swedish Ethical Review Authority, respectively (protocol numbers 2016/131 and 2020-04131). All experiments were carried out in accordance with the approved protocols.

### Generation of gRNAs and Cas9 plasmids

The pSpCas9(BB)-2A-Puro (PX459) V2.0 plasmid was used for the transfection experiments (a gift from Feng Zhang, Addgene plasmid #62988; http://n2t.net/addgene:62988; RRID: Addgene_62988). Guide RNA coding sequences were cloned into PX459 V2.0 using BbsI (#ER1011, Thermo Fisher Scientific), as previously described.^61^ Insertion of the gRNA cassette was verified by Sanger sequencing using the following primer: 5′-*GGC CTA TTT CCC A*TG ATT CCT-3′.

### Transfection and selection

To introduce CRISPR plasmids into human fibroblasts, we used electroporation (P2 Primary Cell 4D-Nucleofector X Kit, #V4XP-2024; Lonza, Basel, Switzerland). One million cells were transfected (program no. CZ-167) with 2 μg of PX459 V2.0 containing either *PSEN1* M146L or scramble gRNA as control. One day after transfection, cells were treated with 1 μg/mL puromycin (#A1113803, Thermo Fisher Scientific) for 24 h, enabling selection of Cas9-2A-Puro-expressing cells from non-expressing cells. Each cell population was further cultured until confluency in the 6-well format was reached. Cells from one well were harvested for DNA extraction, and three additional wells were used to collect conditioned media for ELISA measurements.

### DNA sequencing and bioinformatics analysis

DNA was extracted from human fibroblasts using the PureLink Genomic DNA Mini Kit (Thermo Fisher Scientific) and resuspended in 10 mM Tris-HCl (pH 8.5). We performed a PCR using a high-fidelity DNA polymerase (Phusion Hot Start II High-Fidelity PCR Master Mix, Thermo Fisher Scientific) with intronic primers flanking exon 5 of the *PSEN1* gene (forward: 5′-*TGA CAA CCA CTT GTC AGC CC*-3′; reverse: 5′-*AGA ACA GGG TGG AAA GCA AA*G A-3′). The PCR products were separated on a 1% agarose gel and purified using a column-based precipitation method (PureLink PCR Purification Kit, Thermo Fisher Scientific). The purified PCR products (500–600 bp DNA) were submitted for Sanger sequencing.

For the first round of NGS, targeted exome sequencing was performed for all selected exons, including at least 25 nucleotides surrounding exons, from the fibroblast DNA samples (scramble AG07867, AG08446, and CRISPR-Cas9-treated AG07867, AG08446, and AG07883) using the Life Technologies AmpliSeq sequence enrichment method. This was followed by Life Technologies IonTorrent sequencing. Sequenced gene regions were aligned to the human reference genome (assembly hg19). Targeted exome sequencing with sequence alignment was performed at the Uppsala Genome Center. For the analyzed *PSEN1* M146L mutation region, the sequencing depth was at least 300 times for all included samples. For the second round of NGS, total genomic DNA was extracted from treated and control samples and 1 kb fragments surrounding the editing sites were amplified by PCR. The PCR products were then submitted for NGS-mediated gene-editing analysis (TIGERQ, Lund, Sweden). The samples were treated with NexteraXT (Illumina), and indexed tagmentation libraries were sequenced with 2 × 150 bp paired-end reads.

The percentage of allelic disruption, for both rounds of NGS, was analyzed using the CRISPResso2 software within the Docker containerization system.^27^ Sequencing reads were analyzed with the following parameters for identification of indel formation in the *PSEN1*^*WT*^ and *PSEN1*^*M146L*^ alleles:

--amplicon_seq AAT CTA TAC CCC ATT CAC AGA AGA TAC CGA GAC TGT GGG CCA GAG AGC CCT GCA CTC AAT TCT GAA TGC TGC CAT CAT GAT CAG TGT CAT TGT TGT CAT GAC TAT CCT CCT GGT GGT TCT GTA TAA ATA CAG GTG CTA TAA G, AAT CTA TAC CCC ATT CAC AGA AGA TAC CGA GAC TGT GGG CCA GAG AGC CCT GCA CTC AAT TCT GAA TGC TGC CAT CAT GAT CAG TGT CAT TGT TGT CCT GAC TAT CCT CCT GGT GGT TCT GTA TAA ATA CAG GTG CTA TAA G --amplicon_name WT,M146L --guide_seq GAA CCA CCA GGA GGA TAG TC -amas 60 --min_identity_score 60 -w 1 -c ATC TAT ACC CCA TTC ACA GAA GAT ACC GAG ACT GTG GGC CAG AGA GCC CTG CAC TCA ATT CTG AAT GCT GCC ATC ATG ATC AGT GTC ATT GTT GTC ATG ACT ATC CTC CTG GTG GTT CTG TAT AAA TAC AGG TGC TAT AAG.

Off-target loci were first amplified using custom primers (designed by TIGERQ) and subsequently sequenced and analyzed in the same manner as mentioned above by adjusting amplicon and gRNA sequences to match the target loci.

### ELISA measurements

For ELISA measurements, cells were grown to confluency before they were further cultured for 48 h in FBS-free cell-culture medium. Such conditioned medium was collected and subjected to ELISAs specific for Aβ40 (BNT77-BA27; no. 294-64701) and Aβ42 (BNT77-BC05; no. 292-64501; Wako Pure Chemicals Industries, Osaka, Japan). Both assays were performed according to the manufacturer’s instructions. A total of 200 μL of conditioned medium was analyzed in duplicates from each cell line.

### Cellular viability and detection of ATP levels

Cellular viability was assessed with alamarBlue Cell Viability Reagent (#DAL1025, Thermo Fisher Scientific) following the manufacturer’s instructions. In brief, fibroblasts were seeded into 6-well plates at a density of 100,000 cells/well. After 72 h, the medium was supplemented with 10% alamarBlue reagent, and following a 2 h incubation, the fluorescence (Ex_560_/Em_590 nm_) was measured using the Tecan Infinite M200 Pro plate reader. Detection of ATP levels in cultured fibroblasts was performed with an ATP assay kit (#ab83355, Abcam, Cambridge, UK). The same seeding density and culture time as for the cellular viability experiment was applied. Following the manufacturer’s instructions together with the deproteinization protocol, fluorescence (Ex_535_/Em_587 nm_) was measured using the Tecan Infinite M200 Pro plate reader.

### FRET/FLIM analysis

The FRET/FLIM assay was conducted as previously described.^62^ In brief, non-mutated control, *PSEN1*^*M146L/WT*^, and treated *PSEN1*^*M146L/WT*^ fibroblasts were plated on coverslips in 6-well plates, subsequently fixed with 4% paraformaldehyde, and stained with antibodies that detect the C-terminus (S182, #P7854, Sigma-Aldrich, Darmstadt, Germany; 1:100) and N-terminus (APS11, #ab15456, Abcam; 1:100) of PS1. Alexa 488 (donor) and Alexa 555 (acceptor) fluorophore-conjugated secondary antibodies (Thermo Fisher Scientific) were used to label the PS1 C- and N-termini, respectively. A femtosecond-pulsed Chameleon Ti:Sapphire laser (Coherent, Santa Clara, CA, USA) at 850 nm was used for two-photon fluorescence excitation. The Alexa 488 fluorescence was acquired using 515/530 nm emission filter. The donor fluorophore lifetimes were measured with a high-speed photomultiplier tube (MCP R3809; Hamamatsu, Bridgewater, NJ, USA) and a fast time-correlated single-photon counting acquisition board (SPC-830; Becker and Hickl, Berlin, Germany). The data were analyzed using SPCImage software (Becker and Hickl). The donor fluorophore lifetime (*t*_1_) in the absence of an acceptor served as a negative control. Shortening of the donor fluorophore lifetime (*t*_2_) in the presence of an acceptor fluorophore indicates FRET, i.e., that the two PS1 terminals are in close proximity (<10 nm) of each other and that the protein is functional.^11^ The %FRET efficiency (*E*_FRET_) was calculated using the following equation: *E*_FRET_ = (*t*_1_ − *t*_2_)/*t*_1_. Higher *E*_FRET_ indicates closer PS1 C- to N-terminal proximity and PS1 in tight, pathogenic conformation.

### Cell lysis

Cell-culture medium was thoroughly removed and the cells were lysed in ice-cold lysis buffer (20 mM Tris [pH 7.5], 0.5% Triton X-100, 0.5% deoxycholic acid, 150 mM NaCl, 10 mM EDTA, 30 mM NaPyro) supplemented with a protease inhibitor cocktail (Thermo Fisher Scientific). The lysates were transferred to protein LoBind tubes (Eppendorf, Hamburg, Germany) and incubated for 30 min on ice prior to centrifugation at 10,000 × *g* for 10 min at 4°C. The supernatants were transferred to new tubes and stored at −70°C until analysis.

### Western blot analysis

Protein concentrations of the total cell lysates were measured with a Pierce BCA protein kit (Thermo Fisher Scientific) according to the manufacturer’s instructions. A total of 3 μg of protein was mixed with Bolt LDS Sample buffer and Sample Reducing agent (both from Thermo Fisher Scientific) and incubated for 10 min at 70°C to denature the proteins. Samples were loaded on a Bolt 4%–12% Bis-Tris plus gel and run in Bolt MES SDS running buffer (both from Thermo Fisher Scientific) for 22 min at 200 V. PageRuler Plus Prestained Protein Ladder, 10 kDa to 250 kDa (#26619, Thermo Fisher Scientific), was used for visualization of gel migration, protein size, and orientation. Transfer to a nitrocellulose membrane was performed for 1 h at 10 V in Bolt transfer buffer containing 10% methanol and 0.1% Bolt antioxidant (Thermo Fisher Scientific). Blocking of the membrane was performed in 5% Blotting-Grade Blocker (#1706404, Bio-Rad, Hercules, CA, USA) in 0.1% Tris-buffered saline + Tween (TBS-T) for 1 h on shake at room temperature, prior to overnight incubation with primary antibodies in at 4°C. Antibodies used in the study were rabbit anti-PS1 C-terminal fragment (CTF) (#5643, D39D1, Cell Signaling Technology, Danvers, MA, USA, 1:500) and mouse anti-PS1 N-terminal fragment (NTF) (#823404, Biolegend, San Diego, CA, USA, 1:1,000). Following 20 min washes in TBS-T, the membrane was incubated with horseradish peroxidase (HRP)-conjugated secondary goat anti-rabbit and goat anti-mouse antibodies (1:1,000, Pierce, Thermo Fisher Scientific) in 5% Blotting-Grade Blocker in 0.1% TBS-T for 1 h on shake at room temperature. Development of the membrane was performed with enhanced chemiluminescence (GE Healthcare, Chicago, IL, USA) by using a ChemiDoc XRS with Image Lab Software to visualize the intensity of the immunoreactive bands (Bio-Rad). Quantification of the bands was performed using Fiji software and a calculation process previously described. In brief, rectangles of the same size were used to measure the intensity of all bands of each protein of interest. A background area was also measured, the intensity of which was subtracted from every value (adjusted intensity).

### *In vitro* detection of off-target effects

ONE-seq was performed as previously described.^28^ In brief, the human reference genome (hg19) sequence was informatically searched for closely matched sites (up to six mismatches also including DNA/RNA bulges) to the *PSEN1* gRNA target sequence to generate a ONE-seq library, which was synthesized as an oligonucleotide library (#G7238A, Agilent Technologies, Santa Clara, CA, USA). The synthesized oligonucleotide library was converted to double-stranded DNA by PCR amplification. Cas9 RNP was generated by incubating *in vitro* transcribed *PSEN1* M146L gRNA with purified SpCas9 (#M0386S, New England Biolabs [NEB], Ipswich, MA, USA) at a 2:1 ratio for 10 min at 25°C. Amplified ONE-seq libraries were incubated with Cas9 RNP at a ratio of 10:1 library/RNP for 2 h at 37°C. After *in vitro* cleavage, Illumina sequencing adapters were ligated for 10 min at 25°C using the Quick Ligation Kit from NEB (#M2200S). Adapter-ligated libraries were PCR amplified and subjected to MiSeq sequencing. Sequencing data were analyzed using custom Python code.

Genomic DNA from fibroblasts containing the M146L allele and *in vitro* transcribed *PSEN1* M146L gRNA were used to perform CIRCLE-seq as previously described.^29^ In brief, Cas9 RNP was generated by incubating *in vitro* transcribed *PSEN1* M146L gRNA with purified Cas9 (#M0386S, NEB) for 10 min at room temperature. Fibroblast DNA was circularized and *in vitro* incubated with Cas9 RNP for 1 h at 37°C. After *in vitro* cleavage, DNA ends were A-tailed for 30 min at 30°C and ligated to Illumina sequencing adapters for 1 h at 20°C using the KAPA library preparation kit (#KK8235, KAPA Biosystems, Wilmington, MA, USA) and subjected to MiSeq sequencing. Sequencing data were analyzed using the CIRCLE-seq informatics pipeline.^29^

The gRNAs used in ONE-seq and CIRCLE-seq were prepared by cloning annealed oligonucleotides encoding the *PSEN1* M146L gRNA sequence (*GAA CCA CCA GGA GGA* TAG TC) into MSP3485 (#140082, Addgene, Watertown, MA, USA). The resulting plasmid was linearized by digestion with HindIII. The linearized plasmid was used as an input template for *in vitro* transcription with the T7 RiboMAX Express Large Scale RNA Production System (#P1320, Promega, Madison, WI, USA) according to the manufacturer’s protocol. The *in vitro* transcribed RNA was purified using the MEGAclear Transcription Clean-Up Kit (#AM1908, Thermo Fisher Scientific) according to the manufacturer’s protocol.

### Statistical analyses

Statistical analyses were performed using GraphPad Prism (version 9). Differences between two groups were evaluated for significance with unpaired or paired two-tailed Student’s t test. Comparisons of three or more groups on a single dataset were performed by Kruskal-Wallis one-way analysis of variance (ANOVA) followed by Dunn’s multiple comparison post hoc test. A p value threshold of 0.05 was used for the assessment of statistical significance. Values are shown as mean ± SD or as median (min ÷ max).

## Supplemental information

Document S1. Figures S1–S4 and Tables S1 and S2

Document S2. Article plus supplemental information
