# Supplementary material for: CRISPR-Cas9 treatment partially restores amyloid-β 42/40 in human fibroblasts with the Alzheimer’s disease PSEN1 M146L mutation
Source: Mol Ther Nucleic Acids. 2022 Mar 28;28:450–61. doi: 10.1016/j.omtn.2022.03.022 (PMC9043867; doi:10.1016/j.omtn.2022.03.022)
Supplement: Document S1. Figures S1–S4 and Tables S1 and S2 [file mmc1.pdf]

OMTN, Volume *28*

Supplemental information

CRISPR-Cas9 treatment partially restores amyloid-β 42/40 in human fibroblasts with the Alzheimer's disease *PSEN1* M146L mutation

Evangelos Konstantinidis, Agnieszka Molisak, Florian Perrin, Linn Streubel-Gallasch, Sarah Fayad, Daniel Y. Kim, Karl Petri, Martin J. Aryee, Ximena Aguilar, Bence György, Vilmantas Giedraitis, J. Keith Joung, Vikram Pattanayak, Magnus Essand, Anna Erlandsson, Oksana Berezovska, and Martin Ingelsson

Supplemental Information

Table S1. Characteristics of fibroblasts used in this study

Sample ID	Description	Sex	Age[2]
**AG07867**	Control	Male	51
**AG07869**	Control	Male	28
**F025**	Control	Male	28
**F008**	Control	Male	44
**AG07872**	*PSEN1* M146L carrier/AD[1]	Male	53
**AG07881**	*PSEN1* M146L carrier	Male	17
**AG07883**	*PSEN1* M146L carrier	Male	22
**AG07887**	*PSEN1* M146L carrier	Female	18
**AG07934**	*PSEN1* M146L carrier	Male	33
**AG08446**	*PSEN1* M146L carrier/AD[1]	Male	38

[1]AD=Alzheimer's disease, [2]Age at time of sampling

Table S2. Editing percentages in CRISPR-Cas9-treated fibroblasts

Sample ID	Allele			
	PSEN1^{WT}		*PSEN1*^{M146L}	
	**Unmodified%**	**Modified%**	**Unmodified%**	**Modified%**
**AG07867**	99.67	0.33	NA	NA
**AG07872**	99.59	0.41	35.35	64.65
**AG07881**	99.48	0.52	32.27	67.73
**AG07883**	98.68	1.32	45.43	54.57
**AG07934**	99.32	0.68	47.86	52.14
**AG08446**	98.77	1.23	40.16	59.84

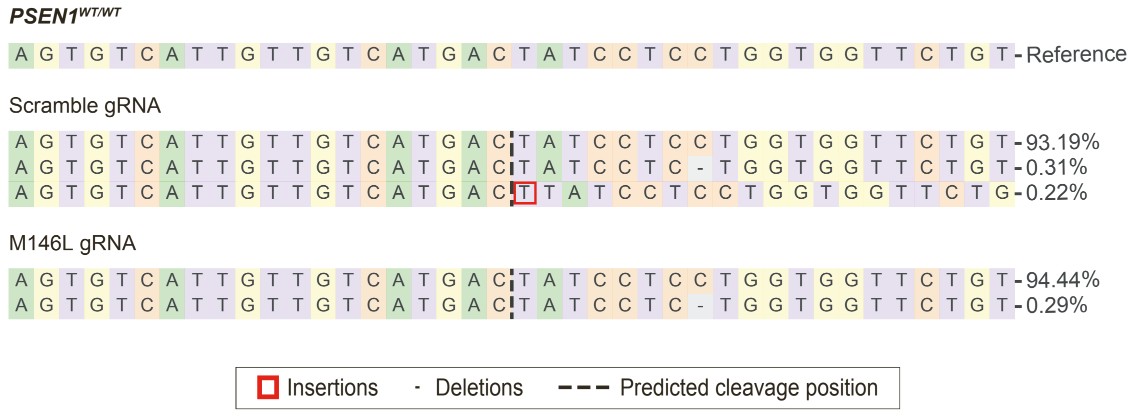

Figure S1. Frequency of identified indels in *PSEN1$^{WT/WT}$* fibroblasts

Visualization of the distribution of identified alleles around the cleavage site after treatment with scramble and M146L gRNA. Nucleotides are indicated by unique colors (A=green; C=red; G=yellow; T=purple). Red rectangles highlight inserted sequences. Horizontal dashed lines indicate deleted sequences. The vertical dashed line indicates the predicted cleavage site.

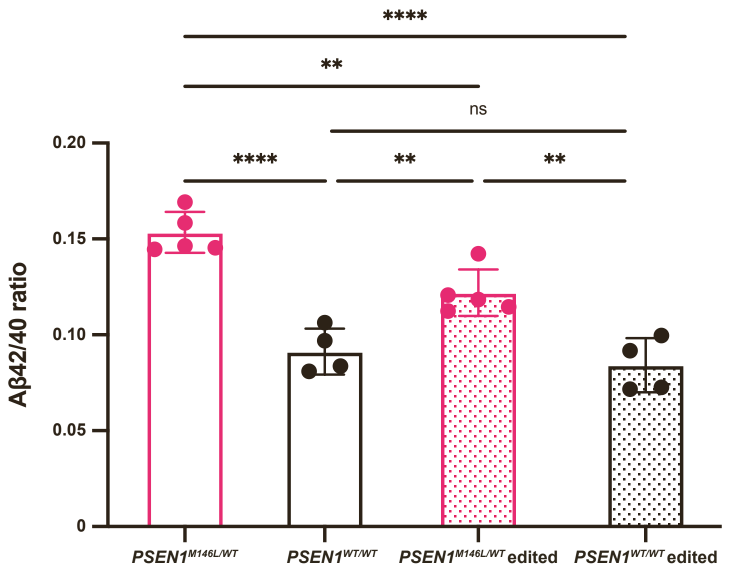

Figure S2. Levels of extracellular Aβ42/40 ratio in CRISPR-Cas9-treated and control *PSEN1$^{M146L/WT}$* and *PSEN1$^{WT/WT}$* fibroblasts

CRISPR-Cas9 treatment significantly reduced the Aβ42/40 ratio in *PSEN1$^{M146L/WT}$* fibroblasts (n = 5) whereas no difference was observed in *PSEN1$^{WT/WT}$* fibroblasts (n = 4). Results are presented as mean±SD; one-way ANOVA with Tukey's multiple comparison post hoc test; ****p<0.0001; **p<0.01.

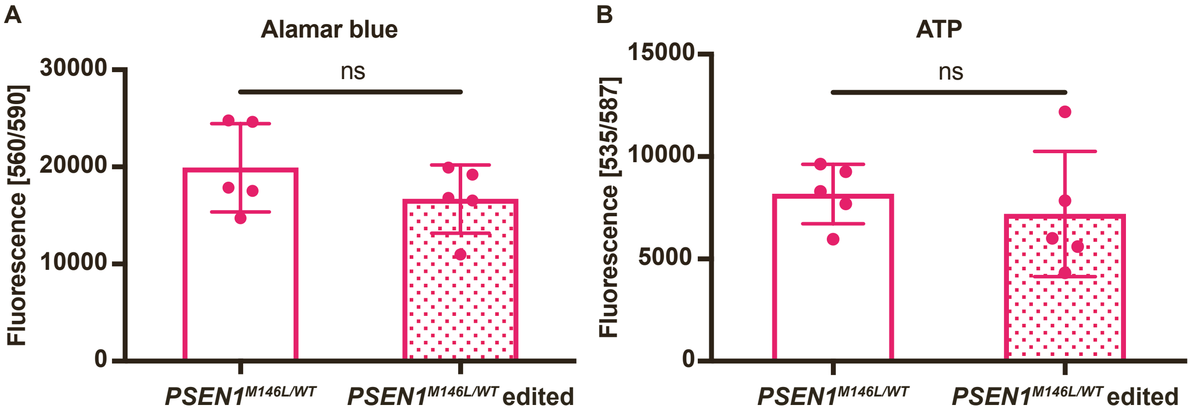

Figure S3. Cellular viability and ATP levels in CRISPR-Cas9-treated and control *PSEN1$^{M146L/WT}$* fibroblasts

(A) CRISPR-Cas9 treatment did not alter cell viability as measured with alamarBlue™ in *PSEN1$^{M146L/WT}$* fibroblasts (n = 5). (B) No difference was observed in ATP levels of *PSEN1$^{M146L/WT}$* fibroblasts (n = 5) before and after CRISPR-Cas9 treatment. Results are presented as mean±SD; unpaired two tailed t-test; ns=non-significant.

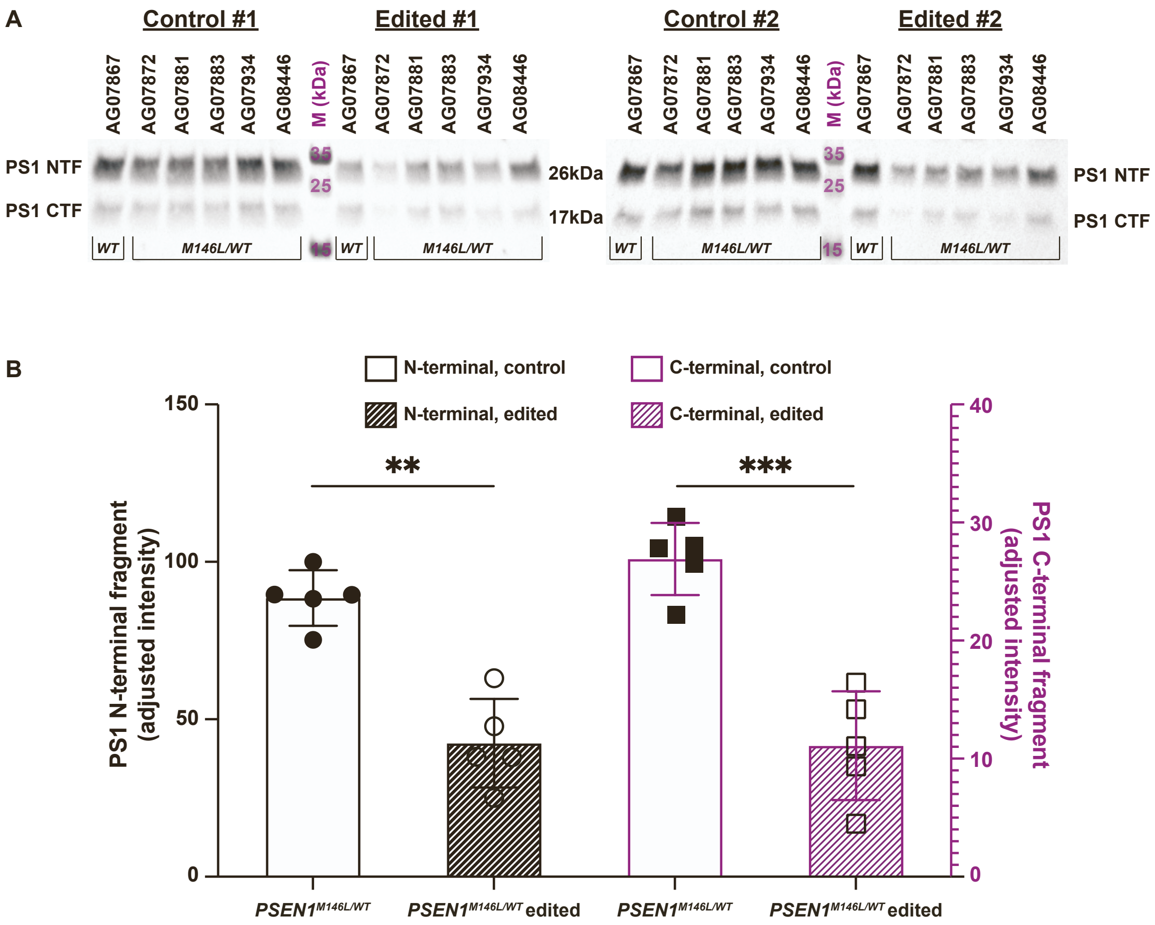

Figure S4. PS1 NTF and CTF levels in CRISPR-Cas9-treated and control *PSEN1*$^{WT/WT}$ and *PSEN1*$^{M146L/WT}$ fibroblasts

(A) Western blot of PS1 NTF (~26kDa) and PS1 CTF (~17kDa) in lysates from *PSEN1*$^{WT/WT}$ (AG07867) and *PSEN1*$^{M146L/WT}$ (AG07872, AG07881, AG07883, AG07934, AG08446) fibroblast samples before and after CRISPR-Cas9 treatment (#1 and #2 are technical replicates of the same samples). (B) Quantification of western blots shown in (A). A significant reduction in both PS1 NTF and PS1 CTF levels was seen overall in *PSEN1*$^{M146L/WT}$ fibroblast samples after CRISPR-Cas9 treatment (n = 5, values refer to adjusted intensity as described in the material and methods section). Results are presented as mean±SD; paired two-tailed t test; ***$p<0.001$; **$p<0.01$.
